# Economic estimation of Bitcoin mining’s climate damages demonstrates closer resemblance to digital crude than digital gold

**DOI:** 10.1038/s41598-022-18686-8

**Published:** 2022-09-29

**Authors:** Benjamin A. Jones, Andrew L. Goodkind, Robert P. Berrens

**Affiliations:** Department of Economics, University of New Mexico, 1 University of New Mexico, MSC 05 3060, Albuquerque, NM 87131 USA

**Keywords:** Energy and society, Environmental economics, Environmental impact, Sustainability

## Abstract

This paper provides economic estimates of the energy-related climate damages of mining Bitcoin (BTC), the dominant proof-of-work cryptocurrency. We provide three sustainability criteria for signaling when the climate damages may be unsustainable. BTC mining fails all three. We find that for 2016–2021: (i) per coin climate damages from BTC were increasing, rather than decreasing with industry maturation; (ii) during certain time periods, BTC climate damages exceed the price of each coin created; (iii) on average, each $1 in BTC market value created was responsible for $0.35 in global climate damages, which as a share of market value is in the range between beef production and crude oil burned as gasoline, and an order-of-magnitude higher than wind and solar power. Taken together, these results represent a set of sustainability red flags. While proponents have offered BTC as representing “digital gold,” from a climate damages perspective it operates more like “digital crude”.

## Introduction

Given rapidly developing blockchain technology and the use of encryption and decentralized, permission-less public ledgers, today’s evolving internet has allowed the emergence of various digitally scarce goods^1^. This digital economy includes nonfungible assets like tokens for various digital media^2^, as well as fungible, divisible assets like the several thousand cryptocurrencies supported by hundreds of exchange platforms^3^. Select digitally scarce goods use production schemes with intensive energy use^4,5^. These include several prominent cryptocurrencies (e.g., Bitcoin, Ether), which to-date are based on highly energy-intensive, competitive tournament-style production schemes known as proof-of-work (POW) mining for providing the encrypted validation in decentralized public ledgers^6,7^.

POW-based cryptocurrencies are a slice of the larger set of blockchain technologies that have disruptively entered global marketplaces over the last decade or more^8^. The production of cryptocurrencies has been relatively decentralized and largely unregulated as they have first gained a foothold and then occupied a larger space^9^. Cryptocurrencies are priced and traded in markets, but often exhibit considerable volatility^10^, and financial anomalies like speculative bubbles^11^, or evidence of price manipulation^12,13^. Yet, various proponents argue that such innovations provide significant value or are especially needed in the developing world (e.g., from providing sustainable new financial goods or mediums of exchange to the underserved^14^, investment diversification^15^, or routes around government corruption^16^). Others question the benefit of such disruptions, and especially so if the new technologies (e.g., POW-type technologies) have intensive energy use, with potentially large social costs from associated carbon emissions^17,18^. Potentially, there may be significant room for learning^19^ and moving to alternative production pathways that use significantly less energy, while still providing the purported benefits^20^. However, achieving net reductions in energy use is inherently challenging, due to redundancies (e.g., number of nodes involved, or the workload of operations) in all types of blockchain technology^21^. Against this backdrop and within broader efforts to mitigate climate change, the policy challenge is creating governance mechanisms for an emergent, decentralized industry, which includes energy-intensive POW cryptocurrencies^22,23^. Such efforts would be aided by measurable, empirical signals concerning potentially unsustainable climate damages.

Taking Bitcoin (BTC) as our focus, this analysis estimates climate damages of mining coins and explores several criteria for signaling when these damages might be unsustainable. First, the trend of estimated climate damages per BTC mined should not be increasing, as the industry matures. Second, per BTC mined, its market price should always exceed its estimated climate damages; i.e., BTC mining should not be “underwater” wherein per unit climate damages are greater than coin market prices for any appreciable period. Third, to contextualize the sustainability of BTC over some chosen time frame, estimated climate damages per coin mined should favorably compare to some reference percentage benchmark of the climate damages per unit market value of other sectors and commodities; e.g., ones that we regulate or consider unsustainable. We offer these measurable criteria for consideration as “red flags” of incipient climate damage from an emerging industry. They signal the need for change (e.g., production alternatives). Absent such change, it may be time to forgo a “business-as-usual” approach and consider collective action (e.g., increased regulation).

### Energy use for mining cryptocurrencies

The proof-of-work (POW) blockchain technology used by Bitcoin (BTC) is energy intensive^5,24^. For context, BTC is a cryptocurrency with a decentralized open-source blockchain whose public ledger began in 2009^25^ and is transacted peer-to-peer without any central authority (e.g., bank or government). Through December 2021, BTC had an approximately $960 billion (US$) market capitalization, and a roughly 41% global market share among all cryptocurrencies ^26^.

POW blockchain technology is energy intensive because new blocks are added to the blockchain through a competitive consensus-driven verification process carried out by individual or pools of “miners.” Miners verify transactions occurring on the blockchain and compete simultaneously to correctly provide a unique transaction identifier, or “hash,” for a block^27^. Miners who are first to verify a given number of transactions and to provide the correct hash identifier are rewarded with new cryptocurrency and a new block is added to the chain^28^.

Providing the correct hash identifier employs enormous amounts of energy due to the decentralized production process, which encourages competition and creates a “winner-take-all” game^27^. As miners across the globe compete, as quickly as possible, to add new blocks to the chain (i.e., by generating guesses of the target hash identifier [“hash rate”]), they employ highly specialized computer equipment and machinery (known as “mining rigs”) that uses significant amounts of electricity to operate competitively^4^. As miners compete with ever more computing power (e.g., as more miners participate in the network, or, as more efficient mining rigs are employed, or both), the overall network hash rate increases, endogenously raising the computational difficulty required to correctly guess the target hash, thereby increasing the overall energy use of mining activity^29^.

## Results

### Bitcoin’s global electricity usage

**Figure 1:**
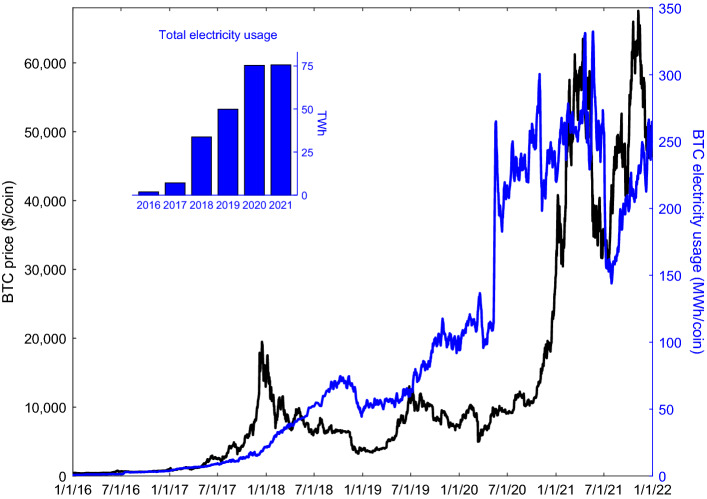
Global 7-days averaged daily electricity usage of mining activity (right axis) and coin exchange price in US$ (left axis) for Bitcoin (BTC). Data from January 1, 2016 to December 31, 2021 shown. Electricity usage is calculated based on network hash rate data downloaded from Blockchain Charts (https://www.blockchain.com/charts) and mining rig efficiency (see Methods section). Prices downloaded from Yahoo! Finance (https://finance.yahoo.com/cryptocurrencies/). All network hash rate and price data are supplied in the Supplementary Data.

Using network hash rate data from January 2016 through December 2021 and data on mining equipment power consumption and efficiency^5,30^, Fig. 1 presents global electricity usage of mining BTC and prices per coin. On the basis of these estimates, in 2020 BTC mining used 75.4 TWh yr^−1^ of electricity, which is more energy than used by Austria (69.9 TWh yr^−1^ in 2020) or Portugal (48.4 TWh yr^−1^ in 2020)^31^. There is a general upward time trend in BTC electricity use and a close correlation between BTC prices and mining energy usage. The decline in BTC exchange prices and mining energy use in the summer of 2021 is likely due in part to China’s banning of financial institutions and payment companies from providing cryptocurrency-related transactions^32^.

Estimates from Cambridge University suggest the majority of electricity used to mine POW cryptocurrencies comes from coal and natural gas, though hydropower use was likely prominent in China until cryptocurrency mining was banned there^32,33^. Globally, it is estimated that 39% of POW mining is powered by renewable energy, meaning that non-renewables, such as fossil fuels, power the majority (~ 61%)^33^. Due to its considerable fossil fuel energy use, cryptocurrency mining contributes to global carbon emissions^30,34^ with associated environmental damages^35^. Goodkind et al.^29^ estimated that in 2018 each $1 (US$) of BTC market value created through mining was associated with $0.49 (US$) in *combined* health and climate damages in the US and $0.37 (US$) in China. Krause and Tolaymat^5^ estimated that BTC, Ether, Litecoin, and Monero coins were responsible for 3–15 million tonnes of CO_2_ emissions over January 2016 to June 2018. For comparison, in 2018, similar amounts of CO_2_ were emitted from Afghanistan (7.44 million tonnes), Slovenia (14.1 million tonnes), and Uruguay (6.52 million tonnes)^36^.

### Climate damages associated with bitcoin mining

**Figure 2:**
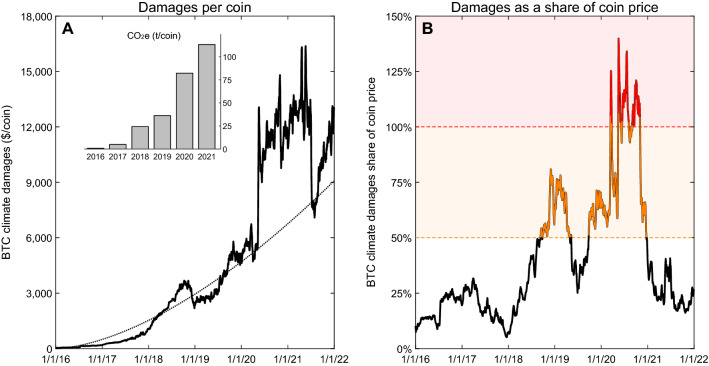
Global estimates of Bitcoin (BTC) mining’s climate damages, CO_2_e emissions, and climate damages as a share of coin price. (**A**) Estimated climate damages ($/coin mined) and CO_2_e emissions (t/coin mined; bar chart) of BTC. A non-linear trend line has been fit to the damages per coin data to illustrate time trends (dotted line). (**B**) Climate damages as a share of the coin’s price for BTC. Values displayed are the 7-days running average. Climate damages per coin mined in (**A**) were divided by the daily market price of the coin and multiplied by 100 to put into percentage terms for calculation in (**B**). $100 t^−1^ damage coefficient used for CO_2_e emissions based on ranges in the peer-reviewed literature. Damages are in US$. Estimates span January 1, 2016 to December 31, 2021. See the Supplementary Data for emissions factors used and the climate damages data.

As mining efforts have increased over time, we estimate steeply increasing CO_2_e (carbon dioxide equivalent) emissions per coin created. Using a global estimate of the location of BTC miners and the local electricity mix, and regional CO_2_e emission coefficients by generation type^37^, a BTC mined in 2021 is responsible for emitting 126 times the CO_2_e as a BTC mined in 2016—increasing from 0.9 to 113 tonnes (t) CO_2_e per coin from 2016 to 2021 (Fig. 2A).

With increasing CO_2_e emissions per coin created, climate damages of producing BTC increased over time (Fig. 2A). Using a $100 t^−1^ damage coefficient for CO_2_e emissions (dollar values in US dollars (US$) unless otherwise noted), commonly referred to as the social cost of carbon (SCC), each BTC created in 2021 resulted in $11,314 in climate damages, on average, with total global damages of all coins mined in 2021 exceeding $3.7 billion. Between 2016 and 2021, total global BTC climate damages are estimated at $12 billion. With rapid price increases in BTC at the end of 2020, climate damages of mining represented 25% of market prices for 2021 (Fig. 2B). This percentage is useful to normalize the scale of externalities to the market price of the product. We offer two potential ranges of concern in Fig. 2B—when the climate damages as a share of the coin price are between 50 and 100% (shown in amber), and when they are > 100% (shown in red). The former would be above those found on average in Goodkind et al.^29^, while the latter represents times when BTC was “underwater” on a per coin basis (i.e., climate damages exceeding the coin’s market price). With much lower prices in 2019 and 2020, BTC climate damages were 64% of market price, on average. For more than one-third of the days in 2020, BTC climate damages exceeded the price of the coins sold. Damages peaked at 156% of coin price in May 2020, suggesting each $1 of BTC market value created in that month was responsible for $1.56 in global climate damages.

By our first sustainability criterion that “the trend of the estimated climate damages per BTC mined should not be increasing, as the industry matures,” BTC fails. There is a clear upward trajectory in per coin estimated climate damages, as seen from the non-linear trend line in Fig. 2A. Rather than declining as the industry matures, each new BTC coin mined is, on average, associated with increasing climate damages.

BTC also fails our second sustainability criterion that “per BTC mined, its market price should always exceed its estimated climate damages.” From Fig. 2B, at multiple periods of time in 2020, BTC climate damages as a share of the coin’s price were greater than 100% (areas indicated in red). BTC was “underwater” at these intervals, meaning that each coin mined produced climate damages exceeding the market price of the coin. Over 2016–2021, BTC was underwater on 6.4% of days, and the damages exceeded 50% of coin price on 30.6% of days.

### What if the social cost of carbon is varied?

One key parameter where we assume a range of values from available evidence is the SCC. For our baseline estimate, we follow Pindyck^38^ in choosing $100 t^−1^. SCC is the estimated present value of monetary damages from emitting an additional tonne of carbon today and monetizes the negative social externalities of carbon emissions^38^. From a policy and regulatory perspective, SCC is a key parameter for evaluating the social costs (i.e., those not considered in the market price) of a high-energy use product or service. Carleton and Greenstone^39^ note the central role of the United States (US) Government’s official SCC estimate in both domestic US and international climate policy. SCC estimation has extensive history in economics^40–42^, and such values are widely used^39^.

However, while analyses that use SCC estimates must make assumptions on its value or range, there is no consensus^38^. There is a growing literature on both estimating the SCC and modeling the optimal SCC for pricing the externality^43^. The current US Government estimated SCC value is $51 t^−1^ CO_2_e in 2020 inflation-adjusted dollars^44^. However, President Biden’s Executive Order #13,990 (January 20, 2021) directed an updating of this value^45^.

Even a select review of recent SCC estimation studies encompasses a broad range of values^38,40,43^. Depending on varying assumptions and approaches, recent empirical studies can easily support a range of values around our SCC baseline coefficient of $100 t^−1^ CO_2_e, from + /−$50 t^−1^ on either side. Thus, to represent some of this variability we use two alternative SCC values to augment the $100 t^−1^ baseline: (i) $50 t^−1^ CO_2_e (essentially equivalent to the 2020 value of the 2010 US Government estimate), and; (ii) $150 t^−1^ CO_2_e.

We re-estimate climate damages of BTC using these alternative SCC values (Supplementary Table 1). The high and low values of the SCC adjust the estimated climate damages proportionally to the baseline value of $100 t^−1^ CO_2_e, and greatly impact the magnitude of the estimated damages. At $150 t^−1^ CO_2_e, BTC climate damages per coin mined averaged $4632 over 2016–2021, compared to $1544 at $50 t^−1^ CO_2_e, versus $3088 at $100 t^−1^ CO_2_e from the results in Fig. 2A. With the high SCC, the climate damages were underwater 17% of the time between 2016 and 2021 (69% of days in 2020), whereas with the low SCC the climate damages were never underwater. Regardless of SCC value, climate damages of BTC mining increased substantially from 2016 to 2021, with a continuing upward trajectory.

### What if mining used more renewable energy?

The CO_2_e emission estimates and climate damages depend, critically, on assumptions of the share of renewable electricity sources used in cryptocurrency mining. Due to the decentralized and anonymized nature of cryptocurrency mining, determining actual energy sources is a challenge and no primary data sources exist^30^. This has led to a range of estimates in the literature. Prior work suggests the share of renewables (e.g., solar, wind, hydropower) used by POW mining processes may vary considerably, from 25.1% of mining’s total electricity use^37^, to 39%^33^ and even up to 73%^46^. Some of the differences in estimates are due to the time periods studied. China, once a large source of global Bitcoin mining that likely used significant amounts of renewable hydropower^30^, banned all cryptocurrency mining in 2021^32^. This appears to have drastically altered the global share of renewables used by Bitcoin miners, resulting in an increased use of fossil fuels^37^. Thus, renewable share estimates before and after the China ban would be expected to be different, and perhaps considerably so. Other differences, such as the methods used to locate miners, assumptions on mining rig efficiency and cooling needs, and assumptions on electricity sources can also drive differences in the range of estimates found in prior work^30,37^.

Given the large ranges found, we expand our analysis with an alternative higher renewable electricity scenario. In this scenario, we increase the share of renewable generation used to mine cryptocurrencies from the baseline of 38.5% (plus 5.2% nuclear power) to a scenario with 50% more renewables (to 57.8% in total plus 5.2% nuclear). This scenario represents a hypothetical situation in which cryptocurrency miners use substantially more renewables than the baseline and a large majority (63%) of electricity from directly carbon free sources (renewables and nuclear combined).

Compared to the baseline renewable share, increasing use of renewables in BTC mining reduces associated climate damages per coin mined (Supplementary Table 2). With a 50% increase in the renewable share, BTC climate damages are approximately two-thirds of the baseline magnitude. Yet, even for this high renewable scenario the climate damages still average 23% of the coin’s price (2016–2021), despite miners only using 37% of their electricity from fossil fuels. Thus, even if BTC miners obtained the majority of their electricity from renewables and directly carbon free sources, there are still large and growing climate damages.

### Comparison to other commodities

**Figure 3:**
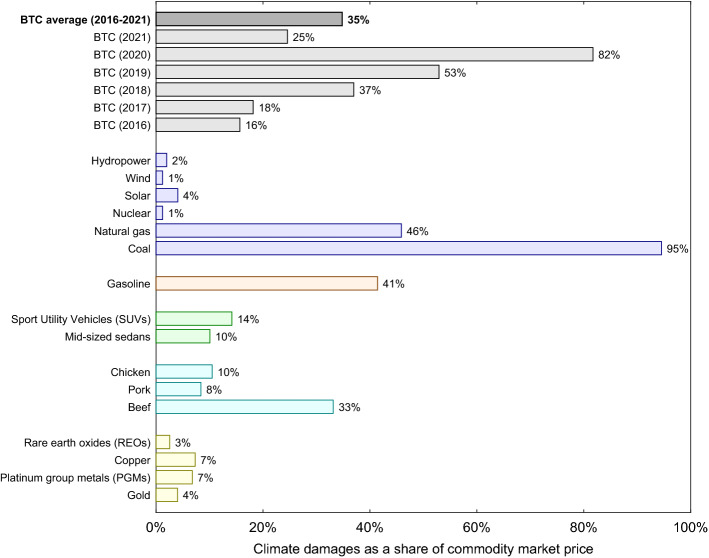
Bitcoin (BTC) mining’s climate damages as a share of coin market price (2016–2021), compared with full lifecycle analysis climate damages as a share of market price for other commodities (for a single year). Damages are expressed in percentage terms (% of market price). BTC climate damages only include energy use and emissions from running mining rigs, and do not include climate damages associated with cooling and manufacturing of mining rigs or other potential sources of carbon equivalent emissions. This makes estimated BTC damages a lower bound compared to the full lifecycle damages for the other commodities shown. Climate damages for the other commodities and economic products shown are calculated using lifecycle estimates from the peer-reviewed literature and US government agencies combined with publicly available price data. All commodity prices and lifecycle climate damage data are in the Supplementary Data.

Recall from Fig. 2B, which showed climate damages per coin market price, that the ratio of BTC damages to price declined from 2020 to 2021. This does not necessarily imply that the POW mining process is sustainable. To contextualize these ratios, we make climate damage comparisons against some other relevant commodities and economic products: (i) electricity generation by source (hydropower, wind, solar, nuclear, natural gas, and coal), (ii) crude oil processed and burned as gasoline, (iii) automobile use and manufacturing (sport utility vehicles (SUVs) and mid-sized sedans), (iv) agricultural meat production (chicken, pork, and beef), and; (v) precious metals mining (rare earth oxides (REOs), copper, platinum group metals (PGMs), and gold). Figure 3 shows climate damages per unit market price (% of price) for BTC compared to lifecycle climate damages of these 16 other commodities.

Climate damages of BTC averaged 35% of its market value (2016–2021), and 58% (2020–2021). This places BTC in the category of other energy intensive or heavily-polluting commodities such as beef production, natural gas electricity generation, or gasoline from crude oil, and substantially more damaging than what we might consider to be more sustainable commodities like chicken and pork production and renewable electricity sources like solar and wind. For solar and wind specifically, their full lifecycle climate damages as a share of their market prices are an order-of-magnitude below those of BTC over 2016–2021. BTC mining also generates climate damages per unit price that are an order-of-magnitude above those generated from the mining of precious metals such as gold, copper, PGMs, and REOs, which all average < 10% per unit market value compared to BTC’s 35% average over 2016–2021. For the specific case of gold, which is considered by some to be an important store of value and a hedge against volatility in stocks, bonds, and the US dollar^47^, BTC’s climate damages are a relative outlier. As a share of gold’s market price, its climate damages average 4%; BTC’s 2016–2021 average climate damages are 8.75 times greater.

Given the high share of climate damages to BTC market price, we ask: “What utilization share of renewable electricity sources would make BTC production similar in climate damage impact to more sustainable commodities?” Our results suggest that if the share of renewable electricity sources for 2016–2021 increased from 38.5 to 88.4% (with additional 5.2% from nuclear)—a 129% increase—the climate damages as a share of coin price for BTC would drop from 35 to 4.0%; similar in magnitude to the climate damages of solar power or gold.

Absent such an extreme increase in the share of renewable electricity used in mining, BTC’s climate damages will remain an outlier compared to more sustainable commodities. Thus, BTC mining presently fails our third sustainability criterion that “estimated climate damages per coin mined should favorably compare to some reference percentage benchmark of the climate damages per unit market value of other sectors and commodities.” Though not as climate damaging as coal electricity generation, BTC mining generates similar damages as gasoline, natural gas generation and beef production, as a share of market prices; none of which would generally be considered sustainable^48,49^.

## Discussion

Digitally scarce goods are likely here to stay, and will bring innovation to a variety of economic dimensions generating value to people. It is important to sort this broader context from the elements of this digital economy that may have particularly significant sustainability and climate concerns (see President Biden’s March 2022 Executive Order on cryptocurrencies for the US^50^). Our focus is on the dominant cryptocurrency, BTC, which uses a highly energy-intensive, competitive POW mining scheme. While society and nations weigh the benefits and costs of various digitally scarce goods, we provide an empirical approach for evaluating BTC sustainability concerns.

We find that for 2016–2021: (i) per coin climate damages from BTC were increasing; (ii) as a share of its market price, BTC climate damages were underwater 6.4% of days, and damages exceeded 50% of the coin price 30.6% of days; and (iii) the average BTC climate damage share was 35% over the period, which falls in the range between beef production and gasoline consumption (as processed from crude oil), but is less than coal electricity generation. BTC’s climate damages per unit market price are roughly an order-of-magnitude higher than wind and solar generation; i.e., it is operating far above any renewable benchmark that might be offered. Taken together, the results represent a set of red flags for any consideration as a sustainable sector (investment or otherwise). While proponents regularly offer BTC as representing a kind of “digital gold”^51,52^, from a climate damages perspective BTC operates more like “digital crude.”

There are a number of important caveats about our offered criteria. First, as to our second criterion, the meaningfulness of our “underwater” benchmark (where the ratio of per coin climate damages as a share of market price not exceed 100%) could be called into question. This exceedance occurs 6.4% of the study period for BTC. While this might be a clear alarm threshold, might it be too weak? Why not 50%, or even staying below 25%? To help consider this, we turn to our third criterion, where we make comparisons to other commodities and sectors. In doing so, staying under a 10% share for an emergent technology might be a preferable sustainability criterion—a level exceeded by BTC 96% of the days in our study.

We highlight that for our comparison commodities, the shares all represent full lifecycle damage estimates, but not for BTC. Thus, BTC shares are deflated in this initial research, ignoring carbon emissions from cooling of mining rigs, rig manufacturing, electronic waste, building construction, etc., where only very preliminary impact estimates are emerging in the literature^35^. A further caveat, with respect to our second and third criteria, relates to accumulating evidence that some cryptocurrency prices may be inflated by significant speculation, and even manipulation (referred to as “crypto washing”) ^13^. Naturally, an inflated price will artificially decrease the estimated climate damages to price ratio. To the extent that artificial price inflation is occurring, the damage ratio with a not-manipulated price may be higher than those presented here. Finally, we have focused strictly on climate damages, but many technology assessments also include health damages from emissions. Thus, for several reasons our sustainability evaluations for BTC are highly conservative.

While not the focus of this paper, an alternative cryptocurrency production process to POW, known as proof-of-stake (POS), could be used to lower the energy use of cryptocurrency mining. POS works by requiring validators to hold and stake coins, with the next block writer on the blockchain being selected at random, with higher odds being assigned to those with larger stake positions^53^. POS, by relying on randomization and validation sharing, does not require significant computational power and therefore uses a fraction of the electricity as POW mining. Ethereum, the second largest cryptocurrency by market capitalization^26^, is scheduled to switch from POW to POS sometime in 2022, lowering its estimated energy use by 99.95%^54^. If Bitcoin, the dominant global cryptocurrency, could also switch from POW to POS, its energy use, and, by extension, its climate damages estimated in this work, would likely become negligible. However, the likelihood of BTC switching to POS seems low at present^55^.

There is no shortage of advocates for digitally scarce goods, and the innovation they offer. Even in the pages of *Nature Climate Change*, Howson^20^ argues: “Remaining overly fixated on the inefficiency of some cryptocurrencies is likely to encourage throwing the blockchain baby out with Bitcoin’s bathwater.” But the danger of path dependence and technological lock-in with an emergent industry^56,57^ supports the argument that POW-based cryptocurrencies, which dominate market share, do indeed merit special attention. Our counterfactuals show that extreme changes would be required to make BTC sustainable (e.g., on the renewable mix). POW-based cryptocurrencies are on an unsustainable path. If the industry doesn’t shift its production path away from POW, or move towards POS, then this class of digitally scarce goods may need to be regulated, and delay will likely lead to increasing global climate damages.

## Methods

### Climate damages of Bitcoin mining

Estimates of climate damages from Bitcoin mining follow methods described in the existing literature in this space^5,29^. The primary estimate of interest is electricity consumption per BTC coin mined (in kWh per coin), as derived from the daily network hash rate of the BTC blockchain^58^; this is the number of calculations on the network in gigahashes per second (GH/s). Using an estimate of average efficiency of BTC mining rigs, in joules (J) per GH, we calculated total electricity consumption (in kWh/day) of the network in Eq. (1), after converting J/s to kilowatts (kW) and multiplying by 24 h per day:

1 $${\text{electricity }}\,\,{\text{consumption}} \left( {\frac{{{\text{kWh}}}}{{{\text{day}}}}} \right) = \,{\text{hash }}\,\,{\text{rate}}\left( {\frac{{{\text{GH}}}}{{\text{s}}}} \right) \times {\text{efficiency}}\left( {\frac{{\text{J}}}{{{\text{GH}}}}} \right) \times \left( {\frac{{\text{s}}}{{\text{J}}}\frac{{{\text{kW}}}}{1000}\frac{{24 \,{\text{h}}}}{{{\text{day}}}}} \right).$$

We calculated total BTC coins mined per day in Eq. (2) using average time in minutes for a block to be added to the blockchain per day^59^ and the miner reward in BTC coins per block:

2 $${\text{coins/day}} = {\text{reward}} \left( {\frac{coins}{{block}}} \right) \times {\text{block }}\,\,{\text{time}} \left( {\frac{blocks}{{{\text{min}}}}} \right) \times \left( {\frac{{24\, {\text{h}}}}{{{\text{day}}}} \cdot \frac{{60 \,{\text{min}}}}{{\text{h}}}} \right).$$

Dividing electricity consumption of the network by the number of coins yields the electricity per coin in Eq. (3):

3 $${\text{electricity}}\,\,{\text{ per}}\,\,{\text{ coin}} \left( {\frac{{{\text{kWh}}}}{{{\text{coin}}}}} \right) = {\text{electricity}}\,\,{\text{ consumption}} \left( {\frac{{{\text{kWh}}}}{{{\text{day}}}}} \right) \div \left( {\frac{{{\text{coin}}}}{{{\text{day}}}}} \right).$$

Multiplying electricity per coin by a global average estimate of the greenhouse gas emission factor (EF) for electricity in the BTC network (in kg CO_2_e/kWh) produces our estimate of emissions per coin in Eq. (4). The emission factors used are provided in the Supplementary Data.

4 $${\text{emissions }}\,\,{\text{per}}\,\,{\text{ coin}} \left( {\frac{{t CO_{2} e}}{coin}} \right) = {\text{electricty}} \left( {\frac{{{\text{kWh}}}}{{{\text{coin}}}}} \right) \times {\text{EF}} \left( {\frac{{kg CO_{2} e}}{{{\text{kWh}}}} \cdot \frac{t}{{1000\, {\text{kg}}}}} \right).$$

Climate damages per coin are calculated as emissions per coin times the SCC (in $/t CO_2_e) in Eq. (5):

5 $${\text{damages}}\,\,{\text{ per}}\,\,{\text{ coin}} \left( {\frac{\$ }{coin}} \right) = {\text{emissions}} \left( {\frac{{t CO_{2} e}}{coin}} \right) \times {\text{SCC}} \left( {\frac{\$ }{{t CO_{2} e}}} \right).$$

Damages as a share of coin price takes the damages per coin and divides by the daily market price of BTC^60^. All estimates of annual or multi-year damages per coin or damages per share of coin price take a daily-coin-generated weighted average across days (i.e., weighted by number of coins generated each day).

Mining rigs improved the efficiency of hash calculations per unit of energy over our study period. For BTC, we calculated annual average rig efficiency from sales data in^30^ for 2016–2018, and then used the efficiency of the popular ANTminer s15 for rig efficiency for 2021. We fit a non-linear relationship (Eq. 6) between this data to compute a declining but flattening rig energy usage per hash for any day in our study period:

6 $${\text{efficiency}} \left( \frac{J}{GH} \right) = 1.3415 \times 10^{9} \exp \left\{ { - 0.00054 \,\,{\text{days}}} \right\}$$

where days is the number of days since 1/1/1900.

Greenhouse gas emissions of electricity generation of the BTC network of miners comes from^37^. We averaged their monthly estimates of global emission factors (kg CO2e/kWh) from September 2019 through August 2021, and applied this average across our study period. The emission factors in^37^ are based on mining pool locations and country and sub-country (China and US) electricity mixes and generation-source-specific emission factors. As sensitivity analyses, we used emission factors from two other sources: (i) from^30^, and; (ii) the US average electricity mix by year using electricity source and generation mix estimates from various US government agencies^61,62^. Results from these analyses are provided Supplementary Table 3 and are qualitatively similar to our baseline results.

### Comparison commodities climate damages

Climate damages from 16 comparison commodities are calculated: electricity generation by source (hydropower, wind, solar, nuclear power, natural gas, and coal); crude oil processed and burned as gasoline; automobile use and manufacturing (sport utility vehicles (SUVs) and mid-sized sedans); agricultural meat production (chicken, pork, and beef), and; precious metals mining (rare earth oxides (REOs), copper, platinum group metals (PGMs), and gold). For each commodity we use estimates of full lifecycle CO_2_e emissions per unit of production, and multiply this by the SCC to obtain climate damages per unit. Climate damages per unit are divided by market price to get damages as a share of commodity value. All commodity price and CO_2_e emissions data per unit production are provided in the Supplemental Data.

For the electricity sector, we used the average lifecycle CO_2_e emissions per kWh electricity generated for the US from the NREL^61^, by source type, and the electricity generation mix by source type for each year from the US EIA^62^. For the market price of electricity, we use the 2016–2021 average retail price across the residential, commercial, industrial, and transportation sectors from the US EIA^63^.

For the agricultural meat sector, we obtained estimates of the lifecycle CO_2_e emissions per head from the FAO^64,65^; for North America (pork), for North America (broilers), for North America (beef). We adjusted for average quantity of meat per carcass to get emissions per kg of meat (pork: 65%, beef: 65%, chicken: 100%) using data from university state extension services^66,67^. The chicken price is per carcass (not per kg of meat) and thus 100% of the carcass is used. Price data are averaged from 2016 to 2020, obtained from the USDA Economic Research Service for pork, beef, and chicken^68^.

For gasoline from crude oil, we use an estimate of the well-to-wheel lifecycle emissions from the literature^69^ and the 2016–2021 average retail price of gasoline from the US EIA^70^.

For vehicles, over a 15-years lifetime, we use estimates of the total cost of ownership and vehicle operation emissions, assuming 14,263 miles annually^71^ based on a 2019 Ford Explorer for a sport utility vehicle (SUV) and a 2019 Toyota Camry for a mid-sized sedan. We add vehicle emissions from fabrication and materials production and extraction using data from the peer-reviewed literature^72^.

For precious metals, annual prices (US$ per troy ounce, US$ per lb, or US$ per kg) for rare earth oxides (REOs), copper, platinum group metals (PGMs), and gold were obtained from the 2021 USGS Mineral Commodity Summaries for 2016–2020^73^. Full lifecycle CO_2_e emissions per unit mass come from^74^ for gold, from the International Platinum Group Metals Association^75^ for PGMs, from^76^ for copper, and from^77^ for REOs.

## Supplementary Information

Supplementary Information.

## Data Availability

All data used in this paper are included in the article and in the Supplementary Information file or are publicly available online as noted.
